# Strangulated inguinal hernia containing ischemic appendices epiploicae

**DOI:** 10.1093/jscr/rjag075

**Published:** 2026-03-22

**Authors:** Shadin Abushara, Ngoud Al Braik, Dawood Mahmood, Sajid Ansari, Ahmed Haidaran

**Affiliations:** General Surgery Department, Galway University Hospital, Newcastle Road, Galway City, H91YR71, Ireland; General Surgery Department, Galway University Hospital, Newcastle Road, Galway City, H91YR71, Ireland; General Surgery Department, Galway University Hospital, Newcastle Road, Galway City, H91YR71, Ireland; General Surgery Department, Galway University Hospital, Newcastle Road, Galway City, H91YR71, Ireland; General Surgery Department, Cork University Hospital, Wilton Street, Cork City, T12DC4A, Ireland

**Keywords:** strangulated inguinal hernia, epiploic appendagitis, appendices epiploicae infarction, rare hernia contents, emergency hernia surgery

## Abstract

A 79-year-old male presented with a two-day history of painful, irreducible left inguinal swelling. Computed tomography imaging indicated a fat-containing hernia with surrounding inflammatory changes. Surgical exploration revealed infarcted appendices epiploicae within the hernia sac. The necrotic tissue was excised, and a tissue-based repair using nylon darning was performed due to contamination. The patient recovered uneventfully and remained well at follow-up.

## Introduction

Inguinal hernias are among the most common general surgical conditions encountered. They are more frequent in males, with a male-to-female ratio of 12: 1 [1]. Complications such as strangulation occur when the vascular supply to the herniated content is compromised, necessitating urgent intervention. While bowel or omentum is typically implicated, strangulation of appendices epiploicae is a rare phenomenon [2]. Documented rare inguinal hernia presentations can be a Littre’s hernia, which is a hernia containing Meckels divertulum or Amyand’s hernia, which is a hernia with the sac containing an appendix [3]. This case presents a very rare strangulated inguinal hernia with a sac containing twisted gangrenous appendices epiploicae, highlighting the diagnostic challenges and surgical management involved.

## Case report

A 79-year-old male patient presented with a 2-day history of constant, painful, irreducible left inguinal swelling. He had initially noticed the swelling 5–6 days earlier, associated with intermittent pain, but delayed seeking medical attention as he continued to pass normal bowel motions. However, when the pain became constant over the preceding two days, he presented to the hospital. On clinical examination, the hernia was tender and extended into the scrotum. The swelling was irreducible, fixed, and without overlying skin changes or scrotal edema. A palpable cord was appreciated. The patient had a background of atrial fibrillation and a right hip prosthesis on long-term antibiotics due to infection.

A computed tomography abdomen and pelvis revealed a left-sided inguinal hernia containing fat, most likely omentum, with associated surrounding fat stranding and free fluid, raising concern for strangulation. The hernia tracked into the visualized left hemiscrotum. There was no evidence of bowel obstruction, pneumoperitoneum, or intra-abdominal collection.

**Figure 1:**
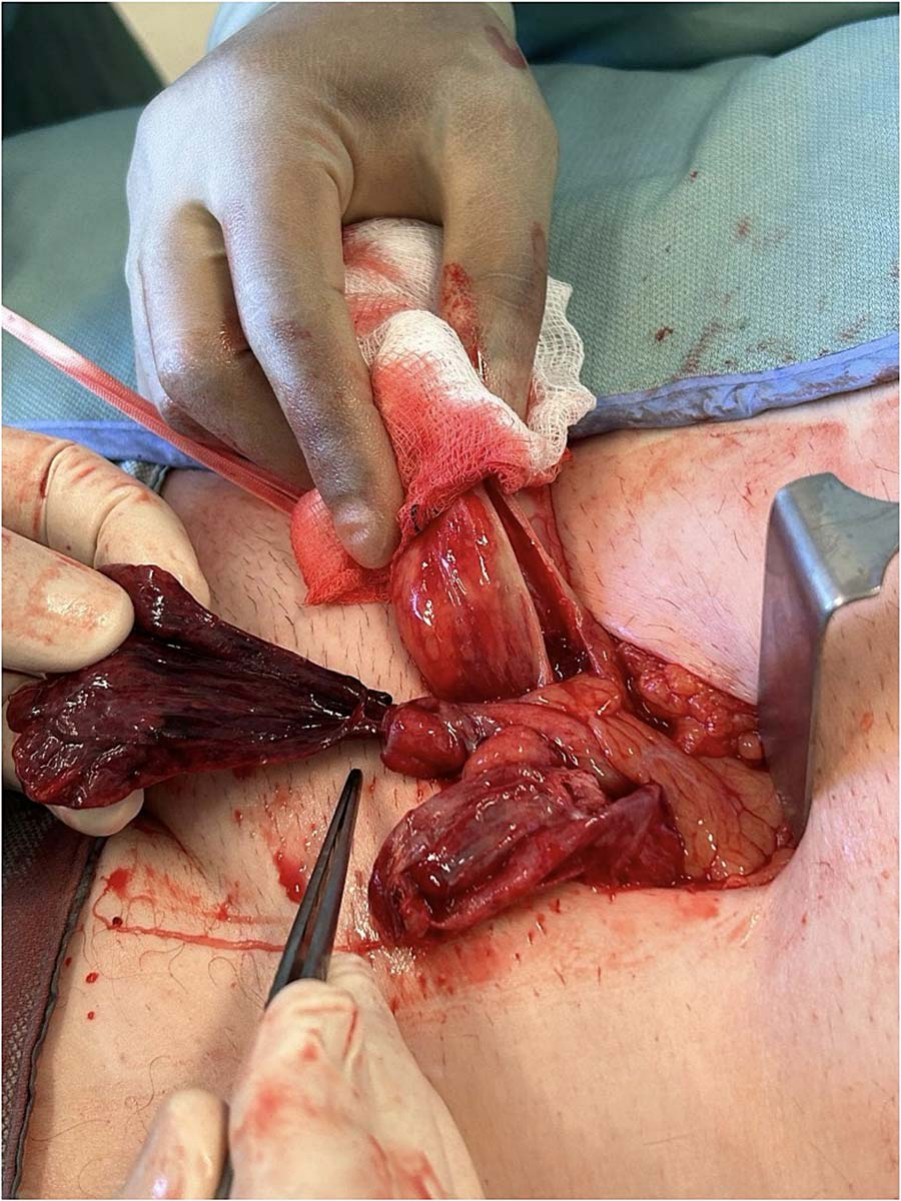
Intraoperative image showing the exposed strangulated appendix epiploica emerging from the left inguinal canal. The tissue appears dark and gangrenous.

**Figure 2:**
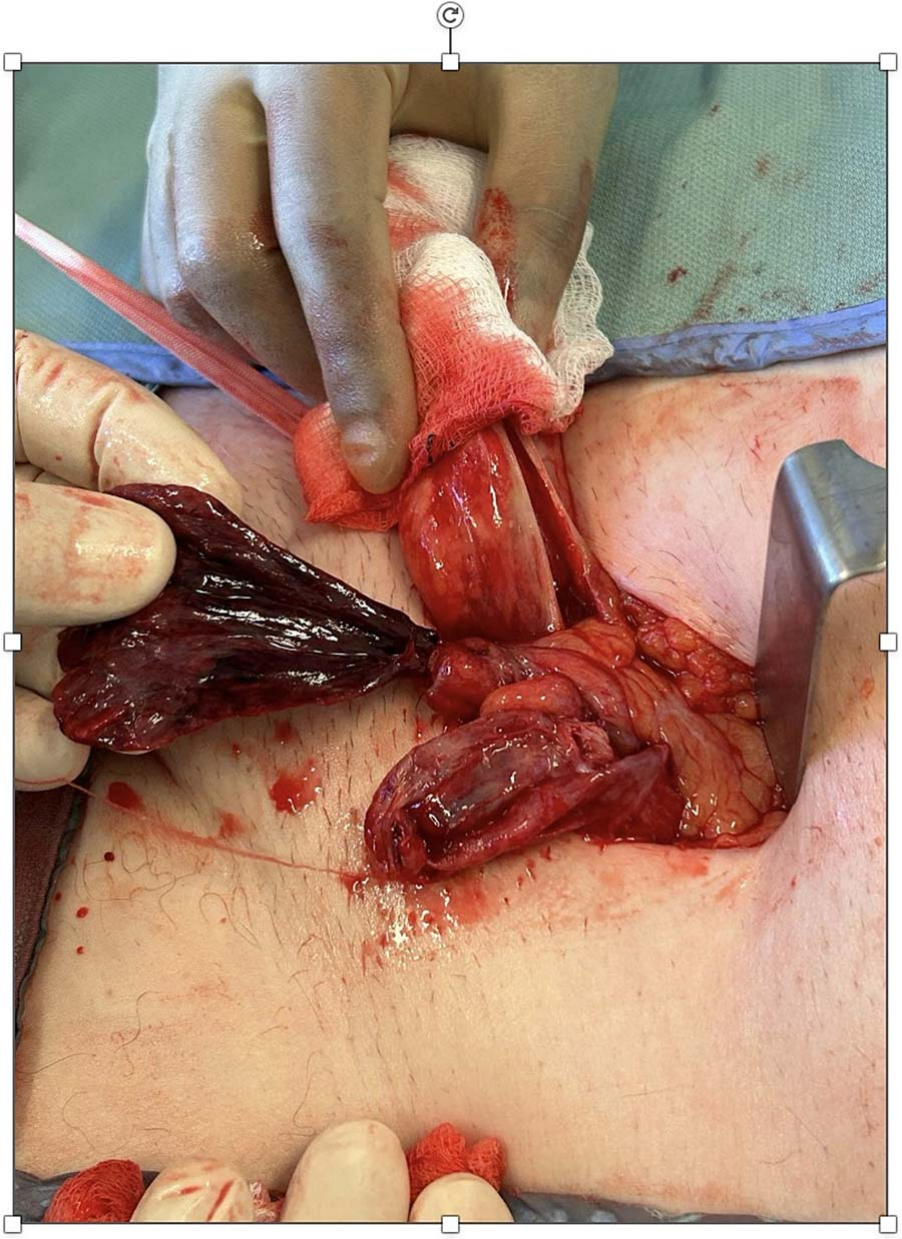
Closer intraoperative view highlighting the thickened, infarcted appendix epiploica and inflamed cord structures following sac exploration.

During surgery, the inguinal canal was opened and found to contain fluid. The cord structures were thickened, edematous, and dark in color. Upon opening (Figs 1 and 2), the hernia sac, a necrotic, twisted structure consistent with a large ischemic appendices epiploicae measuring approximately 6–7 cm, was identified. This was excised and ligated using 2–0 Vicryl. The vas deferens and associated vessels were carefully isolated. There was no sigmoid colon attached to the sac. Due to the presence of contaminated fluid, mesh repair was avoided, and a loop nylon 0–0 darning technique was used for hernia repair.

The patient’s postoperative course was uneventful. He was discharged the following day on a 7-day course of oral co-amoxiclav. At one-week follow-up, he remained well with no evidence of wound infection or hernia recurrence.

Histopathological examination confirmed ischemic appendices epiploicae. Microscopy showed hemorrhagic necrosis of adipose tissue, thick-walled blood vessels, and acute inflammation. No malignant changes were noted. The hernial sac was composed of mesothelial-lined fibro-adipose tissue with central fibrous content and vascular changes.

## Discussion

Appendices epiploicae are fat-filled peritoneal outpouchings along the colon, typically ranging from 0.5 to 5 cm. Torsion or venous thrombosis of an appendix epiploica may cause infarction and present clinically as localized abdominal pain, often mimicking other pathologies such as diverticulitis or incarcerated hernia [4]. Strangulation of an appendix epiploica within an inguinal hernia is exceedingly rare. It is hypothesized that preexisting hernia sacs may intermittently contain and eventually trap these mobile structures, especially in older individuals with lax peritoneal attachments [5]. CT imaging may aid in diagnosis by identifying a fat-density lesion within the hernia sac; however, ischemic changes are more readily appreciated intraoperatively. Due to the contaminated field in our case, prosthetic mesh was avoided in favor of a tissue-based repair, consistent with European Hernia Society guidelines [6].

## Conclusion

This case illustrates a rare and important differential for irreducible inguinal hernias, strangulated appendices epiploicae. Prompt surgical exploration remains crucial to prevent further complications. A high index of suspicion, thorough interpretation of imaging, and appropriate intraoperative decision-making are essential. Awareness of atypical hernia contents can improve operative outcomes and surgical planning.
